# Significance of soluble triggering receptor expressed on myeloid cells-1 elevation in patients admitted to the intensive care unit with sepsis

**DOI:** 10.1186/s12879-016-1893-4

**Published:** 2016-10-12

**Authors:** P. E. Charles, R. Noel, F. Massin, J. Guy, P. E. Bollaert, J. P. Quenot, S. Gibot

**Affiliations:** 1 Service de Réanimation Médicale, Hôpital Bocage Central, C.H.U. Dijon, 14 rue Gaffarel, 21000 Dijon, France; 2 Laboratoire d’Immunologie, C.H.U. Brabois, Vandoeuvre-les-Nancy, France; 3 Laboratoire d’Hématologie, Plateau Technique de Biologie, C.H.U. Dijon, Rue Angélique de Coudray, 21000 Dijon, France; 4 Service de Réanimation Médicale, Hôpital Central, C.H.U, 29 avenue du Maréchal de Lattre de Tassigny, 54035 Nancy Cedex, France; 5 Medical Intensive Care Unit, Hôpital Bocage Central, Centre Hospitalier et Universitaire de Dijon, 14, rue Gaffarel, 21000 Dijon, France

## Abstract

**Background:**

Among septic patients admitted to the intensive care unit (ICU), early recognition of those with the highest risk of death is of paramount importance. Since clinical judgment is sometimes uncertain biomarkers could provide additional information likely to guide critical illness management. We evaluated the prognostic value of soluble Triggering Receptor Expressed by Myeloid cells 1 (sTREM-1), procalcitonin (PCT) and leucocyte surface expression of CD64.

**Methods:**

This was a prospective cohort study, which included 190 septic patient admitted to the ICU in two hospitals. Blood samples for biomarker measurements were obtained upon admission and thereafter. The Simplified Acute Physiology Score (SAPS) II and the Sequential Organ Failure Assessment (SOFA) score were calculated. The primary outcome was all-cause death in the ICU.

**Results:**

The mortality rate reached 25.8 %. The best predictive value of the three biomarkers was obtained with baseline sTREM-1, although clinical scores outperformed this. Accuracy was greater in patients without prior exposure to antibiotics and in those with proven bacterial infection. Adding sTREM-1 levels to SAPS II increased its specificity to 98 %. The soluble TREM-1 level, core temperature and SAPS II value were the only independent predictors of death after adjustment for potential confounders. A decrease in sTREM-1 with time was also more pronounced in survivors than in non-survivors.

**Conclusions:**

sTREM-1 was found to be the best prognostic biomarker among those tested. Both baseline values and variations with time seemed relevant. Although SAPS II outperformed sTREM-1 regarding the prediction of ICU survival, the biomarker could provide additional information.

**Electronic supplementary material:**

The online version of this article (doi:10.1186/s12879-016-1893-4) contains supplementary material, which is available to authorized users.

## Background

Sepsis remains a leading cause of death worldwide, especially in the intensive care unit (ICU) setting [1]. It is currently accepted that improving the outcome of critically ill patients with sepsis relies mainly on the adequacy and the timeliness of key interventions such as administering appropriate antibiotics and sufficient amounts of fluid, especially the sickest ones [2].

It is therefore mandatory to accurately assess the severity of the acute illness in such patients. Severity scores based on the assessment of underlying disease and organ failure have been derived from large studies [3]. However, these large cohorts included patients without sepsis. In addition, the interest of repeated clinical assessments has not been validated with such scoring systems, and one should consider only the worst values of the physiological and biological parameters within the first 24 h following ICU admission. As a result, the Simplified Acute Physiology Score (SAPS) II is not theoretically available before day 2 and finally of limited value in clinical practice. In contrast, the Sequential Organ Failure Assessment (SOFA) score, which is easier to calculate since it relies solely on daily organ dysfunction assessment, could be more suitable. In addition, it was first evaluated in septic patients [4, 5]. However, as organ failure is the end-stage complication of sepsis, it would be useful to predict it before it becomes clinically obvious, in order to prevent or at least to attenuate it whenever possible. In addition, clinical judgment may lack objectivity, thus leading to wrong evaluations and potentially inappropriate interventions. Moreover, the administration of innovative therapies is thought to provide the greatest benefit if given early to the potentially sickest septic patients.

In addition to the clinical evaluation, biomarkers provide a unique but only theoretical opportunity to predict the risk of bad outcomes reliably and promptly in patients with sepsis. Since the host inflammatory response is of paramount importance, measuring some of its most relevant mediators as well as surrogates within various body fluids including plasma has been proposed as a promising way to improve the management of such patients. Among these biomarkers, procalcitonin (PCT) and the soluble Triggering Receptor Expressed by Myeloid cells 1 (sTREM-1) have been shown to exhibit good diagnostic accuracy for bacterial sepsis [6–8]. More recently, we showed that the CD64 leucocyte index measured upon ICU admission was even more accurate [9].

The prognostic value of these biomarkers, however, remains to be clearly established and compared with relevant clinical scores. Actually, although it is tempting to believe that the same biomarker could be both a reliable diagnosis tool for sepsis and a powerful outcome predictor, none of those mentioned above has demonstrated these abilities within the same cohort of patients.

We therefore assessed the predictive value of PCT, sTREM-1 and the PMN CD64 index, with regard to the risk of a bad outcome in a large cohort of ICU septic patients included in a prospective observational study that aimed primarily to evaluate their diagnostic accuracy.

## Methods

The methodology has already been extensively described elsewhere [9].

### Study population

Briefly, the approval of the institutional review board and written informed consent were obtained before inclusion. All consecutive patients newly hospitalized in two French medical intensive care units (Nancy and Dijon) were prospectively enrolled in the study. There were no exclusion criteria.

### Data collection

On admission to the ICU, the following items were recorded for each patient: age; sex; severity of underlying medical condition stratified according to the criteria of McCabe and Jackson; SAPS II score [10]; Sepsis-related Organ Failure Assessment (SOFA) score (range, 0 to 24, with scores for each organ system [respiration, coagulation, liver, cardiovascular system, central nervous system, and kidney] ranging from 0 [normal] to 4 [most abnormal]) [4]; and the reason for admission to the ICU. The following baseline variables were also recorded at inclusion: body temperature; leucocyte count; ratio of the partial pressure of arterial oxygen to the fraction of inspired oxygen (Pao_2_/Fio_2_); presence of shock, defined as systolic arterial pressure lower than 90 mmHg with signs of peripheral hypoperfusion or need for continuous infusion of vasopressor or inotropic agents; and the use of previous antimicrobial therapy. The length of the ICU stay and ICU deaths were also recorded.

Two intensivists retrospectively reviewed all of the medical records pertaining to each patient and independently classified the diagnosis as no infection, sepsis, severe sepsis, or septic shock at the time of admission, according to established consensus definitions [11]. Only patients with sepsis, severe sepsis, or septic shock were kept for the present study.

### Measurement of Neutrophil CD64 index, and plasma levels of Procalcitonin, and sTREM-1

Within 12 h after admission and enrolment in the study, 5 mL of whole heparinized blood was drawn. Sampling was repeated on days (D) 2,3,5,7,10,14,21, and 28, provided the patient was still in the ICU. The expression of CD64 on neutrophils and monocytes was measured by quantitative flow cytometry using the Leuko64TM assay (Trillium Diagnostics, LLC, Brewer, ME). The sample preparation and flow cytometer setup were based on the manufacturer’s instructions. Index calculations were performed using Leuko64 QuantiCalc software (Trillium Diagnostics, LLC, Brewer, ME) [12]. Flow cytometry was performed within 12 h after blood sampling. The whole procedure took less than 2 h. The reproducibility of measurements was excellent with a coefficient of variation lower than 5 %.

Procalcitonin concentrations were measured using an immunoassay with a sandwich technique and a chemiluminescent detection system, according to the manufacturer’s protocol (LumiTest, Brahms Diagnostica, Berlin, Germany).

Plasma concentrations of sTREM-1 were measured by ELISA using the Quantikine kit assay (RnD Systems, MN, USA) according to the manufacturer’s recommendations. All analyses were performed in duplicate. Inter and intra-assay coefficients of variation were lower than 7 %.

### Clinical endpoints

The outcome of the included patients was assessed according to the two following endpoints: all-cause death in the ICU and an increasing SOFA score between day 1 and day 3 as early surrogates for deteriorating organ function. Given the fact that late mortality may be caused rather by secondary infections or comorbidities than by sepsis itself, biomarkers’ predictive value regarding the risk of death before day-14 was also evaluated.

### Statistical analysis

Descriptive results for continuous variables were expressed as means (±SD) or medians (IQR) depending on the normality of their distribution as assessed by the Kolmogorov-Smirnov test. Variables were tested for their association with the outcome (i.e., death in the ICU or increasing SOFA scores between D1 and D3) using the Pearson *χ* ^2^ test for categorical data and the Mann-Whitney *U* test for numerical data. Receiver-operating-characteristic (ROC) curves were constructed to illustrate various cut-off values of soluble TREM-1 and PCT. The sensitivity, specificity, positive and negative likelihood ratios and their confidence intervals were calculated (19). These values were calculated for the cut-off that represented the best discrimination as derived from the Youden index (J = max [sensitivity + specificity-1]).

To account for wide distributions of data and potential nonlinear associations with outcomes, if mean baseline values were found to be significantly different between survivors and non-survivors, biomarker values were transformed into quartiles based on their distribution. The corresponding Kaplan-Meier curves were then constructed in order to compare ICU survival between the different quartiles through a time-dependent analysis, using the log-rank test.

We also evaluated the value of PCT and sTREM-1 in predicting the outcome as independent factors using a Cox model. Any covariate with univariate significance of *p* < 0.10 was eligible for inclusion in the model.

Finally, we compared PCT and sTREM-1 with the clinical scoring systems usually used in critically ill patients upon ICU admission (i.e., SAPS II and SOFA) and with serum lactate concentration, regarding their ability to predict the outcome. Several combinations were also tested.

Subgroup analyses were conducted in patients with proven infection (i.e., microbiologically documented) and in those who were not given antibiotics within the 48 h preceding ICU admission.

Statview software (Abacus Concepts, Berkeley CA) and Prism (Graphpad®) were used for the analyses. A two-tailed *p* < 0.05 was considered significant.

## Results

The whole data set is available as supplementary material (Additional file 1).

### Baseline characteristics of the study population

Among the 379 patients included in both ICUs during the study period, 190 were deemed infected (130 [68.4 %] with septic shock), of whom 49 (25.8 %) died in the ICU (Table 1). As expected, severe sepsis and septic shock were the main admission diagnoses. Most of the patients did not have an ultimately or rapidly fatal underlying disease according to MacCabe classification. Immunosuppression was reported in 13.7 % of the included patients.

**Table 1:** Patient baseline characteristics and septic episode description according to all-cause ICU mortality

	Overall (*n* = 190)	Survivors (*n* = 141)	Non survivors (*n* = 49)	*p*
Age (years)	60.6 (16.6)	58.8 (17.1)	65.7 (13.8)	0.01
SAPS II (points)	52.6 (21.5)	45.7 (17.2)	73.2 (19.9)	<0.01
Gender. male (N. [%])	109 (57.4)	80 (56.7)	29 (59.2)	0.76
McCabe (N. [%])				0.37
Non fatal	107 (66.9)	97 (68.7)	30 (61.2)	
Ultimately fatal (<5 years)	41 (21.6)	31 (22.0)	10 (24.9)	
Rapidly fatal (<6 months)	22 (11.6)	13 (9.2)	9 (18.4)	
Immunosuppression (N. [%])	26 (13.7)	18 (12.8)	8 (16.3)	0.53
Main admission diagnosis				0.05
Respiratory distress (N. [%])	23 (12.1)	21 (14.9)	2 (4.1)	
Shock (N. [%])	107 (56.3)	75 (53.2)	32 (65.3)	
Severe sepsis (N. [%])	21 (14.3)	19 (13.5)	2 (4.1)	
Neurologic failure (N. [%])	22 (11.6)	16 (11.3)	6 (12.2)	
Miscellaneous (N. [%])	17 (8.9)	10 (7.1)	7 (14.3)	
Septic episode description				
Core temperature (°C)	37.4 (1.2)	37.5 (1.0)	37.1 (1.8)	0.07
Mechanical ventilation (N. [%])	134 (70.5)	88 (62.4)	46 (93.9)	0.02
Septic shock (N. [%])	130 (68.4)	85 (60.3)	45 (91.8)	0.02
SOFA (points)	9.0 (5.2)	7.6 (4.6)	13.0 (4.9)	<0.01
Infection source				0.03
Lung (N. [%])	91 (47.9)	68 (48.2)	23 (46.9)	
Urinary tract (N. [%])	27 (14.2)	26 (18.4)	1 (2.0)	
Intra-abdominal (N. [%])	21 (11.0)	15 (10.6)	6 (12.2)	
Skin and soft tissue (N. [%])	11 (5.8)	7 (5.0)	4 (8.2)	
Primary bacteremia (N. [%])	9 (4.7)	4 (2.8)	5 (10.2)	
Miscellaneous (N. [%])	31 (16.3)	21 (14.9)	10 (20.4)	
Bacteremia (N. [%])	47 (24.9)	33 (23.6)	14 (28.6)	0.49
Isolated pathogen				0.13
Gram-positive (N. [%])	56 (29.6)	44 (31.4)	12 (24.5)	
Gram-negative (N. [%])	43 (22.7)	35 (25.0)	8 (16.3)	
Mixed (N. [%])	6 (3.2)	2 (1.4)	4 (8.2)	
Miscellaneous (N. [%])	9 (4.8)	7 (5.0)	2 (4.1)	
None (N. [%])	75 (39.7)	52 (37.1)	23 (46.9)	
Antibiotics started before ICU admission (N. [%])	57 (30.0)	43 (30.5)	14 (28.6)	0.80

*SAPS* simplified acute physiologic score, *SOFA* sequential organ failure assessment, *ICU* intensive care unit

### Septic episode description

As shown in Table 1, pneumonia was the most frequent cause of sepsis in our cohort. Most of the patients required mechanical ventilation as well as vasopressors upon admission. The mean SOFA score was 9.0 (5.2), reflecting the severity of organ failure. Cultures proved sterile in almost 40 % of the patients since antibiotics had been given prior to ICU admission in 30 % of them. Otherwise, gram-positive bacteria were more frequently isolated than gram-negative bacteria. Bacteremia was detected in around one quarter of the patients.

### Outcomes

All cause ICU mortality was 25.8 % in the study population. “Early” death (i.e., before day-14) occurred in 45 patients. The mean length of stay was 9.5 day (5.0 [1–109]). The mean duration of mechanical ventilation was 6.4 days (2 [0–90]). In addition, the SOFA score remained stable or worsened within the first 48 h of sepsis management in 54 (28.4 %) patients.

### Predictive value of biomarker baseline values regarding ICU mortality

**Fig. 1:**
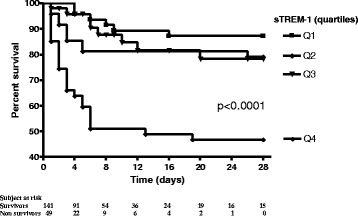
Survival in the ICU according to the sTREM-1 admission level quartile. sTREM-1: soluble triggering receptor expressed on myeloid cells-1; ICU: intensive care unit

In the first set of analyses, the respective predictive value of each biomarker was assessed by univariate analysis. While the CD64 index was not associated with the outcome of the patients, sTREM-1 and PCT elevation was greater in patients with a bad outcome than in those without (Table 2). This was especially true and statistically significant for sTREM-1. After transformation into quartiles, the 4th quartile of sTREM-1 was significantly and markedly associated with a poorer outcome (Table 2, Fig. 1).

**Table 2:** Biomarkers and other potentially relevant measurements on ICU admission according to all-cause ICU mortality

	Survivors (*n* = 141)	Non survivors (*n* = 49)	*p*
Sepsis biomarkers			
PCT (pg/L)	9.1 (32.7)	19.4 (37.4)	0.57
sTREM-1 (ng/L)	671.0 (514.8)	1148.4 (825.3)	<0.01
sTREM-1 quartiles			<0.01
1^st^ quartile (N. [%])	40 (28.2)	7 (14.3)	
2^nd^ quartile (N. [%])	39 (27.4)	9 (18.4)	
3^rd^ quartile (N. [%])	40 (28.2)	8 (16.3)	
4^th^ quartile (N. [%])	22 (16.2)	25 (51.0)	<0.01
Neutrophils CD64 index	3.1 (2.8)	3.1 (3.1)	0.92
Other			
Leukocytes (cells/mm^3^)	12200 (11100)	14500 (15375)	0.72
Neutrophils (cells/mm^3^)	10620 (9010)	13940 (13205)	0.47
Platelets (cells/mm^3^)	152000 (170500)	105000 (145250)	0.05
Lactate (mmol/L)	2.1 (1.9)	4.4 (7.7)	<0.01
Lactate quartiles			
1^st^ quartile (N. [%])	40 (29.0)	7 (14.3)	
2^nd^ quartile (N. [%])	39 (28.3)	8 (17.0)	
3^rd^ quartile (N. [%])	38 (27.5)	9 (18.4)	
4^th^ quartile (N. [%])	21 (15.2)	25 (54.3)	<0.01

*PCT* procalcitonin, *sTREM-1* soluble triggering receptor expressed on myeloid cells-1

Continuous data are presented as mean (standard deviation) and mediane (interquartile range)

**Fig. 2:**
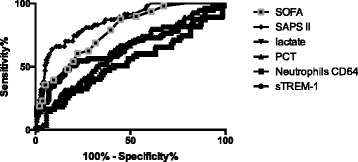
Overall accuracy of various biomarkers and clinical scores regarding ICU survival of septic patients. PCT: procalcitonin; sTREM-1: soluble triggering receptor expressed on myeloid cells-1; SAPS: simplified acute physiologic score; SOFA: sequential organ failure assessment; ICU: intensive care unit

However, the overall predictive values of sTREM-1 and PCT elevation were quite weak according to the AUROCC calculation (0.64 [0.54–0.74] and 0.62 [0.53–0.71]; 95 % CI, respectively) (Tables 3, 4 and 5, Fig. 2). Optimal cut-off values were then determined. Although the PPV was low, the NPV was found to be greater than 80 % for both biomarkers (Table 3). Interestingly, sTREM-1 prognostic value was improved if early mortality was considered, since AUROCC reached 0.75 (0.66–0.84), while PCT performance remained low (0.62 [0.53–0.72]). Lactate elevation performed no better than sTREM-1, whenever overall or early-mortality was considered.

**Table 3:** Clinical performance of biomarkers and clinical scoring systems in predicting all-cause ICU mortality

	AUROCC [95 % CI]	Se (%) [95 % CI]	Sp (%) [95 % CI]	PPV (%) [95 % CI]	NPV (%) [95 % CI]	Positive LR [95 % CI]	Negative LR [95 % CI]
sTREM-1 (>954.4 ng/L)	0.64 [0.54–0.74]	54.5 % [40.5–68.0]	78.0 % [70.3–84.5]	49.2 % [40.5–54.6]	81.5 % [70.3–92.4]	2.48 [1.36–4.39]	0.58 [0.37–0.85]
PCT (>11.1 ug/L)	0.62 [0.53–0.71]	66.7 % [52.5–78.9]	55.8 % [47.1–64.2]	37.1 % [28.3–42.5]	81.0 % [67.1–91.4]	1.51 [0.99–2.20]	0.60 [0.33–1.01]
Lactate (>3.2 mmol/L)	0.71 [0.62–0.80]	61.2 % [46.2–74.8]	78.3 % [70.4–84.8]	50.0 % [37.7–61.1]	84.8 % [70.4–92.0]	2.82 [1.56–4.92]	0.49 [0.30–0.76]
SAPS II (>67.5)	0.86 [0.80–0.91]	63.8 % [48.5–77.3]	91.3 % [85.3–95.4]	71.4 % [56.6–90.2]	88.1 % [81.3–90.9]	7.34 [3.30–16.80]	0.40 [0.24–0.60]
SOFA D1 (>7.5)	0.78 [0.71–0.85]	87.5 % [74.7–95.2]	55.1 % [46.4–63.7]	40.8 % [35.5–45.3]	92.6 % [74.7–100]	1.95 [1.39–2.62]	0.23 [0.07–0.54]

*AUROCC* area under receiver operating characteristics curve, *Se* sensitivity, *Sp* specificity, *PPV* positive predictive value, *NPV* negative predictive value, *LR* likelihood ratio, *PCT* procalcitonin, *sTREM-1* soluble triggering receptor expressed on myeloid cells-1, *SAPS* simplified acute physiologic score, *SOFA* sequential organ failure assessment, *ICU* intensive care unit

**Table 4:** Clinical performance of some biomarkers combinations in predicting all cause ICU mortality

	AUROCC [95 % CI]	Se (%) [95 % CI]	Sp (%) [95 % CI]	PPV (%) [95 % CI]	NPV (%) [95 % CI]	Positive LR [95 % CI]	Negative LR [95 % CI]
sTREM-1 (>954.4 ng/L) & PCT (>11.1 ug/L)	0.64 [0.54–0.73]	38.2 % [25.4–52.3]	89.4 % [83.1–93.9]	58.3 % [35.6–73.2]	78.7 % [75.6–85.4]	3.59 [1.50–8.57]	0.69 [0.51–0.90]
sTREM-1 (>954.4 ng/L) & Lactate (>3.2 mmol/L)	0.62 [0.53–0.72]	29.1 % [17.6–42.9]	95.7 % [91.0–98.4]	49.2 % [29.7–72.5]	83.8 % [79.7–86.2]	6.84 [1.95–26.80]	0.74 [0.58–0.90]

*AUROCC* area under receiver operating characteristics curve, *Se* sensitivity, *Sp* specificity, *PPV* positive predictive value, *NPV* negative predictive value, *LR* likelihood ratio, *PCT* procalcitonin, *sTREM-1* soluble triggering receptor expressed on myeloid cells-1, *ICU* intensive care unit

**Table 5:** Clinical performance of sTREM-1 combined with either SAPS II or SOFA score values in predicting all cause ICU mortality

	AUROCC [95 % CI]	Se (%) [95 % CI]	Sp (%) [95 % CI]	PPV (%) [95 % CI]	NPV (%) [95 % CI]	Positive LR [95 % CI]	Negative LR [95 % CI]
sTREM-1 (>954.4 ng/L) & SAPS II (>67.5)	0.68 [0.59–0.78]	38.8 % [25.2–53.8]	98.6 % [95.0–99.8]	91.3 % [58.8–100]	80.3 % [79.3–83.3]	27.30 [5.08–261.50]	0.63 [0.48–0.78]
sTREM-1 (>954.4 ng/L) & SOFA D1 (>7.5)	0.70 [0.61–0.80]	55.1 % [40.2–69.3]	85.8 % [78.9–91.1]	57.4 % [41.9–72.2]	84.6 % [77.8–89.8]	3.88 [3.20–29.70]	0.57 [0.41–0.76]

*AUROCC* area under receiver operating characteristics curve, *Se* sensitivity, *Sp* specificity, *PPV* positive predictive value, *NPV* negative predictive value, *LR* likelihood ratio, *sTREM-1* soluble triggering receptor expressed on myeloid cells-1, *SAPS* simplified acute physiologic score, *SOFA* sequential organ failure assessment, *ICU* intensive care unit

In contrast, better performances were achieved with the SAPS II and SOFA scores as compared with biomarkers. This was especially true for SAPS II (AUROCC = 0.86 [0.80–0.91]; 95 % CI), with a positive LR reaching 7.34 ([3.30–16.80]; 95 % CI).

Combinations of sTREM-1 and PCT on the one hand, and sTREM-1 and blood lactate on the other hand were then evaluated (Table 4). The overall performance of each biomarker was not improved. Indeed, lactate elevation in addition to sTREM-1 improved the specificity of the latter, but decreased the sensitivity, thereby resulting in an unchanged AUROCC.

The predictive accuracy of sTREM-1 combined with either SAPS II or SOFA scores was also tested (Table 5). Interestingly, adding sTREM-1 to the SAPS II score improved the accuracy of the clinical score, through increased specificity and an enhanced positive LR. However, since the sensitivity was reduced, the overall accuracy of SAPS II for the prediction of death was decreased according to the AUROCC.

In addition, the accuracy of the biomarkers was tested in two clinically relevant subsets of patients, those with proven infection and those with no antibiotherapy prior to ICU admission. Interestingly, the performance of sTREM-1 was enhanced in both groups since the AUROCC reached 0.74 ([0.62–0.86]; 95 % CI) and 0.75 ([0.66–0.85]; 95 % CI), respectively (Additional file 2: Tables S1 and S2). In contrast, the ability of PCT to predict outcomes was not improved in these groups.

In a second set of analyses, a multivariate model was built in an attempt to assess the discriminatory power of sTREM-1 and PCT in predicting outcomes. The relevant variables likely to influence the outcome according to the findings of the univariate analysis were then entered into the model, as described in the methods section. Strikingly, sTREM-1 was found to be an independent predictor of death in the ICU (Table 6). Only core temperature and the SAPS II score also remained strong predictors of outcomes in our model. Conversely, neither PCT elevation nor the lactate level at D1 remained associated with a bad outcome. Similar findings were obtained when including sTREM-1 quartiles into the model (Additional file 2: Table S3).

**Table 6:** Independent predictors of all-cause ICU mortality

	Hazard ratio	95 % CI	*p*
sTREM-1	1.001	1.000–1.002	<0.01
SAPS II	1.039	1.024–1.053	<0.01
Core temperature	0.773	0.629–0.948	0.01

*sTREM-1* soluble triggering receptor expressed on myeloid cells-1, *SAPS* simplified acute physiologic score, *CI* confidence interval

### Predictive value of daily variations of biomarkers regarding ICU death

**Fig. 3:**
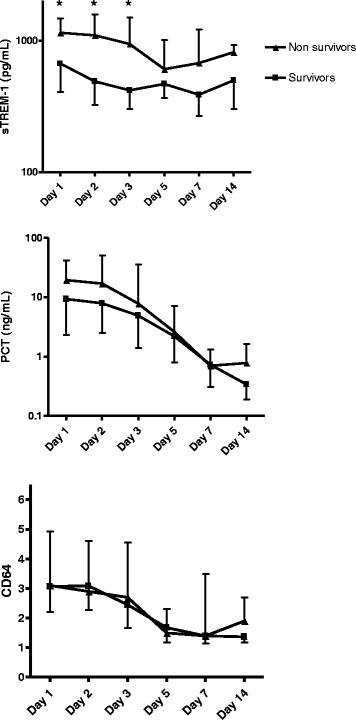
Variations with time of sTREM-1, PCT and PMN CD64 index according to ICU survival of septic patients. PCT: procalcitonin; sTREM-1: soluble triggering receptor expressed on myeloid cells-1; ICU: intensive care unit

In addition to one single baseline value, variations of biomarkers over time could be helpful in predicting the outcome of patients. We therefore assessed to what extent daily variations in sTREM-1, PCT and CD64 were related to death in the ICU. As expected, and in accordance with the above-mentioned findings, sTREM-1 kinetics was found to differentiate between survivors and non-survivors in the ICU (Fig. 3). In contrast, no statistically significant difference was found regarding this endpoint for either PCT or CD64 variations.

### Predictive value of biomarker baseline values regarding clinical worsening

The ability of blood lactate, sTREM-1 and PCT to predict the risk of clinical worsening within the first 48 h of sepsis management as compared with the daily SOFA score was then evaluated (Table 7). Although the overall accuracy as assessed through the AUROCC was weak for all three, the sensitivity and NPV of both sTREM-1 and PCT reached almost 90 %, using threshold values of 568.8 ng/L and 3.9 μg/L, respectively. A blood lactate level rising above the 3.1 mmol/L threshold was found to be more sensitive but less specific than the tested biomarkers, with an NPV and PPV of 75.8 and 50.0 %, respectively. However, the combination of either sTREM-1 or PCT with the blood lactate level did not perform better than either marker alone (data not shown).

**Table 7:** Clinical performance of biomarkers in predicting SOFA score increase within the first 48 h of sepsis management in the ICU

	AUROCC [95 % CI]	Se (%) [95 % CI]	Sp (%) [95 % CI]	PPV (%) [95 % CI]	NPV (%) [95 % CI]	Positive LR [95 % CI]	Negative LR [95 % CI]
sTREM-1 (>568.8 ng/L)	0.66 [0.56–0.76]	86.5 % [71.2–95.5]	45.8 % [35.6–56.3]	38.1 % [31.4–42.1]	89.8 % [69.7–100]	1.60 [1.10–2.18]	0.29 [0.08–0.81]
PCT (>3.9 ug/L)	0.61 [0.51–0.71]	88.2 % [72.5–96.7]	36.5 % [26.9–46.9]	33.0 % [27.1–33.1]	89.7 % [66.2–100]	1.39 [0.99–1.82]	0.32 [0.07–1.02]
Lactate (>3.1 mmol/L)	0.60 [0.51–0.68]	43.0 % [32.8–53.7]	76.6 % [66.7–84.7]	50.0 % [40.2–64.7]	75.8 % [60.1–76.3]	1.84 [0.98–3.44]	0.78 [0.56–1.01]

*AUROCC* area under receiver operating characteristics curve, *Se* sensitivity, *Sp* specificity, *PPV* positive predictive value, *NPV* negative predictive value, *LR* likelihood ratio, *PCT* procalcitonin, *sTREM-1* soluble triggering receptor expressed on myeloid cells-1, *SOFA* sequential organ failure assessment, *ICU* intensive care unit

## Discussion

Accurately evaluating the severity of acute illness in septic patients on admission to an ICU is challenging. It usually relies on a combination of various clinical and biological data, including the assessment of organ failure, as well as knowledge of underlying disease(s). Although of paramount importance, clinical judgment might be biased given the need for prompt decision-making. Indeed, having an objective overview of a patient’s risk of death remains a matter of concern. Calculating clinical scores is fastidious and could remain subjective. Measuring biomarkers could overcome these drawbacks.

The main findings of the present study were the following: (i) the PMN CD64 index had no prognostic value despite its promising accuracy regarding the diagnosis of sepsis; (ii) the predictive value of sTREM-1 regarding the risk of death was greater than that of PCT, especially when considering early mortality. In addition, among the biomarkers tested, sTREM-1 was an independent predictor of death and sTREM-1 kinetics looked different between survivors and non survivors. Nonetheless, both biomarkers were outperformed by clinical scores; (iii) the combination of sTREM-1 and SAPS II offered the best accuracy for predicting ICU survival in our cohort; (iv) none of the indicators tested were valuable tools for reliably predicting clinical worsening within the first 48 h of the ICU stay.

Several studies have aimed to evaluate biomarkers including sTREM-1 as prognostic factors in critically ill patients with sepsis. However, despite interesting findings, it remains unclear whether they are of any value or not (Table 8).

**Table 8:** Summary of the main clinical studies evaluating sTREM-1 prognostic value

	Gibot et al. 2005 [16]	Giamarellos-Bourboulis et al. 2006 [19]	Zhang et al. 2011 [23]	Jeong et al. 2012 [15]	Su et al. 2012 [24]	Li et al. 2014 [13]	Ravetti et al. 2015 [17]	Our study
Nb. of Patients	63	90	52	63	130 (100 with sepsis)	102	40 with cancer	190
Median Age	61	Unknown	Unknown	63.7	58.9	63	67	60.6
Unit	ICU	ICU	ICU	ER (and then ICU)	ICU	ICU	ICU	ICU
Severity of sepsis	Septic shock (53 %)	Severe sepsis + Septic shock (70 %)	Severe sepsis + Septic shock (71.1 %)	Severe sepsis + Septic shock (100 %)	Severe sepsis + Septic shock (64 %)	Sepsis, Severe sepsis and Septic shock	Severe sepsis + Septic shock (100 %)	Septic shock (68.4 %)
Site of Infection	Miscellaneous	VAP	Miscellaneous	Miscellaneous	Miscellaneous	Miscellaneous	Miscellaneous	Miscellaneous
Overall Mortality	33 % (ICU)	36.7 %	30.7 % (D28)	25.4 % (D28)	43 % (D28)	41.2 % (D28)	In cancer patients:	25.8 % (ICU)
							34.7 % (ICU)	
							40 % (D28)	
SAPS II	53 (21)	Unknown	Unknown	Unknown	Unknown	Unknown	Unknown	52.6 (21.5)
APACHE 2	Unknown	Unknown	Unknown	Unknown	13.4 (6.1)	Unknown	In cancer patients 19.9 (5.1)	Unknown
SOFA D0	936 (3.1)	Unknown	Unknown	Unknown	7.8 (4.4)	Unknown	6.2 (2.7)	9 (5.2)
Type of sample	- sTREM1	- Serum	- Serum	- Serum	- Serum	- Serum	- Serum	- Serum
		- sTREM1	- sTREM1	- sTREM1	- sTREM1	- sTREM1	- sTREM1	- sTREM1
		- ELISA	- ELISA	- ELISA	- ELISA	- ELISA	- ELISA	- ELISA
Median sTREM1 level on admission (pg/ml)							In Cancer Patients	
Survivors	154	Unknown	193.4	182.4	Unknown	161.95	848	671
Non survivors	94 (*p* = 0.02)	Unknown	240.2 (NS)	514.1 (*p* = 0.001)	Unknown	320 (*p* < 0.001)	558 (NS)	1148 (*p* < 0.01)
sTREM1 cut-off value (pg/ml)	180 (baseline)					252.05 (baseline)		954.4 (baseline)
Sensitivity	86 %					85.7 %		54.5 %
Specificity	70 %					75.7 %		78 %
AUROCC	0.74					0.856		0.64
PPV						70.6 %		49.2 %
NPV						88.2 %		81.5 %
Best Relevant Prognostic Predictor (associated with sTREM1)	-sTREM1 baseline	- sTREM1/IL-6 baseline ratio	None (sTREM1 increase between D1 and D14 was NS)	- Log (sTREM1) baseline	None	sTREM1 (baseline > 252.05)	-sTREM1 value on D2 for ICU mortality. AUROCC = 0.69	sTREM1 (baseline value > 954.4) + SAPS II (>67.5)
Other relevant Predictors	- SOFA baseline	- TNF alpha baseline	- SOFA score baseline and evolution	- ScVO2 baseline			- sTREM1 value on D1 for D28 mortality. AUROCC = 0.75	
		- IL-6 baseline		- SAPS II	- SOFA	- PCT (baseline > 10.6 ng/ml)	- Days of MV	- SAPS II
		- IL-10/IL-6 baseline ratio			- sCD163	- SOFA baseline > 6.5	- Use of corticosteroids	- Core Temperature

*ICU* Intensive Care Unit, *ER* Emergency Room, *PPV* Predictive Positive Value, *NPV* Negative Predictive Value, *AUROCC* area under receiver operating characteristics curve, *sTREM-1* soluble triggering receptor expressed on myeloid cells-1, *PCT* procalcitonin, *MV* mechanical ventilation, *SOFA* sequential organ failure assessment, *SAPS* simplified acute physiologic score, *NS* not significant, *ELISA* enzyme-liked immuno-assay

TREM-1 is a member of the immunoglobulin superfamily of receptors that is specifically expressed on the surface of neutrophils and monocytes. The primary role of all TREM is both the tuning and integration of multiple signals rather than the direct initiation of an inflammatory response. Soluble TREM-1 is the soluble form of TREM-1, which is up-regulated when the host innate immune system is exposed to infectious invaders. Any sustained increase in the sTREM-1 level indicates that the overall expression of TREM-1 is continuously rising, along with the release of larger amounts of pro-inflammatory mediators. Thereafter, any further increase in sTREM-1 suggests a protracted inflammatory response generally related to a poor clinical outcome.

To our knowledge, this study is the largest to date to evaluate the prognostic interest of measuring sTREM-1 in septic patients. Concerning the predictive value of baseline sTREM-1 levels, our findings are in accordance with those obtained by Li et al., who showed in 102 ICU patients that day-1 sTREM-1 concentrations yielded an AUROCC of 0.85 regarding the risk of death at day-28 [13]. In this study, PCT was also showed a good predictive value. The far higher mortality rate reported in the Li et al. cohort than in ours (41.2 vs. 25.8 %, respectively) may account for the greater accuracy in predicting death. Moreover, the number of patients with antibiotic exposure prior to biomarker measurement was quite high in our study, as was the number of patients with negative cultures. These characteristics probably diminished the predictive value of sTREM-1 [14]. Finally, Jeong et al. also found that the sTREM-1 concentration on admission was the best biomarker regarding the short-term prognosis in patients presenting with severe sepsis, outperforming baseline blood lactate levels [15].

Altogether, however, these results are apparently conflicting if compared with the data previously obtained by our group in 63 septic patients, among whom more than half presented with shock, that the lower the sTREM-1 baseline level, the poorer the outcome [16]. Similar findings were obtained recently in a small cohort of cancer patients. Indeed, Ravetti et al. showed that levels of sTREM-1 over time were higher in survivors than in non-survivors [17]. This may be subsequent to the dual significance of plasma levels of sTREM-1. Basically, it may reflect the overall expression of TREM-1, including the membrane anchored as well as the soluble form. Then, high levels might be deleterious for the host since they could result in an overwhelming inflammatory response. Conversely, the release of large amounts of sTREM-1 could be protective through the neutralization of yet unknown TREM-1 ligands likely to amplify the host inflammatory response [18]. An anti-inflammatory effect of sTREM-1 could then be expected as suggested by findings made in a small cohort of patients with sepsis related to ventilator-associated pneumonia [19, 20]. Moreover, as suggested by previous works, elevated sTREM-1 does not necessarily reflect TREM-1 gene expression [21]. In addition, one could speculate that depending on the immunoassay, either one or more isoforms of sTREM-1, with or without its shed form, were detected, thus accounting for such a discrepancy. Finally, TREM-1 expression on immune cell surfaces is known to be highly time-dependent, but also variable according to the pathogen involved and the source of infection [14]. Differences may exist regarding these points in the different cohorts mentioned above and account for these apparently conflicting findings. However, the present findings may be more representative since a larger number of patients were included in two distinct ICUs.

In addition, we should admit that several other studies failed to demonstrate any interest of sTREM-1 as a predictor of prognosis. For example, Phua et al. found that baseline sTREM-1 was a poor predictor of death in the ICU, as did Zhang et al. [22, 23]. Procalcitonin performed even better in the former study. One larger study published by Su et al. showed similar results, although the sTREM-1 level was probably helpful in diagnosing sepsis, and in differentiating between sepsis, severe sepsis and septic shock [24].

To overcome the above-described issues regarding the interpretation of a single sTREM-1 value, serial measurements are of potential interest. As previously reported, we showed herein that an early decrease in sTREM-1 levels was associated with a better outcome in the ICU [15, 16, 23, 25]. The high rate of previous exposure to antibiotics (around 30 %) in our cohort may account for the lack of correlation between PCT time-course and the outcome. Actually, we and others have previously shown in large cohorts of critically ill patients that a decrease in PCT levels within the first 72 h of sepsis management was closely related to the outcome [26, 27].

Finally, we compared biomarkers with clinical scores since they are still considered the “gold-standard” for predicting the outcome of critically ill patients [4, 10, 28]. It is worth noting that in our study the AUROCC achieved with both SOFA on admission and SAPS II was found to be greater than that for sTREM-1. However, adding sTREM-1 to SAPS II improved its specificity, since it reached 98 %, and showed a positive likelihood ratio of 27.3. In addition, the baseline sTREM-1 level remained an independent predictor of death in the ICU as did the SAPS II score, after adjustment for potential confounders, whereas neither lactate levels nor the SOFA score did. We could then consider that measuring sTREM-1 upon admission to the ICU, provides relevant information regarding the severity of sepsis in addition to clinical data.

There are, however, some limitations. First, our cohort was small, thus precluding the external validity of our findings. Second, sTREM-1 levels were not routinely measured since it relies on one ELISA assay that so far has not been automated. Finally, one can argue that given the large proportion of patients with negative bacterial cultures, non-septic inflammatory states were included in our study. However, this could be easily explained by the fact that prior exposure to antibiotics before ICU admission was quite frequent, thus reflecting “real life” conditions.

## Conclusions

In this cohort of critically ill patients with sepsis, sTREM-1 was found to be the best prognostic biomarker among those tested including PCT. Although SAPS II outperformed sTREM-1 regarding the prediction of ICU survival, the biomarker could provide additional information. Accordingly, our findings emphasize the fact that reliable tools to diagnose sepsis such as PCT are not necessarily essential to predict the prognosis [29].

Nevertheless, further studies are needed to determine to what extent measuring sTREM-1 upon ICU admission in septic patients could be useful for the decision-making process and for patients selection in the setting of clinical trials. Moreover, TREM-1 could be considered a potential therapeutic target in septic patients [30].

## Additional files

Additional file 1: Whole data set used for statistical analysis evaluating prognostic value of biomarkers and severity scores. (XLSX 129 kb)
Additional file 2: Table S1. Clinical performance of biomarkers in predicting all-cause ICU mortality in the subset of patients with proven infection (*n* = 115). **Table S2.** Clinical performance of biomarkers in predicting all-cause ICU mortality in the subset of patients without antibiotic prior to ICU admission (*n* = 133). **Table S3.** Independent predictors of all-cause ICU mortality. (DOCX 98 kb)

## Abbreviations

AUROCC: Area under the operating characteristics curve
CI: Confidence interval
ICU: Intensive care unit
LR: Likelihood ratio
NPV: Negative predictive value
PCT: Procalcitonin
PMN: Polymorphonuklear
PPV: Positive predictive value
SAPS: II Simplified acute physiology score
Se: Sensitivity
SOFA: Sequential organ failure assessment
Sp: Specificity
sTREM-1: Soluble triggering receptor expressed by myeloid cells 1
