# Supplementary material for: Significance of soluble triggering receptor expressed on myeloid cells-1 elevation in patients admitted to the intensive care unit with sepsis
Source: BMC Infect Dis. 2016 Oct 12;16:559. doi: 10.1186/s12879-016-1893-4 (PMC5059941; doi:10.1186/s12879-016-1893-4)
Supplement: Additional file 2: Table S1. — Clinical performance of biomarkers in predicting all-cause ICU mortality in the subset of patients with proven infection (n = 115). Table S2. Clinical performance of biomarkers in predicting all-cause ICU mortality in the subset of patients without antibiotic prior to ICU admission (n = 133). Table S3. Independent predictors of all-cause ICU mortality. (DOCX 98 kb) [file 12879_2016_1893_MOESM2_ESM.docx]

|  | AUROCC  [95% CI] | Se (%)  [95% CI] | Sp (%)  [95% CI] | PPV (%)  [95% CI] | NPV (%)  [95% CI] | Positive LR [95% CI] | Negative LR [95% CI] |
| --- | --- | --- | --- | --- | --- | --- | --- |
| **sTREM-1**  **(>949.4)** | 0.74  [0.62-0.86] | 68.2%  [40.5-68.0] | 75.3%  [70.3-84.5] | 49.2%  [40.5-54.6] | 81.5%  [70.3-92.4] | 2.48  [1.36-4.39] | 0.58  [0.37-0.85] |
| **PCT**  **(>46.3 ug/L)** | 0.54  [0.42-0.66] | 88.0%  [68.9-97.4] | 26.7%  [17.8-37.4] | 37.1%  [28.3-42.5] | 81.0%  [67.1-91.4] | 1.20  [0.99-2.20] | 0.60  [0.33-1.01] |

**Table S1.** Clinical performance of biomarkers in predicting all-cause ICU mortality in the subset of patients with proven infection (n = 115).

AUROCC: area under receiver operating characteristics curve; Se: sensitivity; Sp: specificity; PPV: positive predictive value; NPV: negative predictive value; LR: likelihood ratio; PCT: procalcitonin; sTREM-1: soluble triggering receptor expressed on myeloid cells-1; ICU: intensive care unit.

|  | AUROCC  [95% CI] | Se (%)  [95% CI] | Sp (%)  [95% CI] | PPV (%)  [95% CI] | NPV (%)  [95% CI] | Positive LR [95% CI] | Negative LR [95% CI] |
| --- | --- | --- | --- | --- | --- | --- | --- |
| **sTREM-1**  **(>954.4)** | 0.75  [0.66-0.85] | 68.6%  [50.7-83.1] | 77.5%  [68.0-85.4] | 52.2% [38.6-63.2] | 87.3% [76.6-96.2] | 3.05 [1.58-5.7] | 0.40 [0.20-0.72] |
| **PCT**  **(>13.7 ug/L)** | 0.60  [0.49-0.70] | 64.7% [46.5-80.2] | 58.2% [47.8-68.0] | 34.9% [25.1-43.3] | 82.6% [67.9-96.6] | 1.55 [0.89-2.50] | 0.61 [0.29-1.12] |

**Table S2.** Clinical performance of biomarkers in predicting all-cause ICU mortality in the subset of patients without antibiotic prior to ICU admission (n = 133).

AUROCC: area under receiver operating characteristics curve; Se: sensitivity; Sp: specificity; PPV: positive predictive value; NPV: negative predictive value; LR: likelihood ratio; PCT: procalcitonin; sTREM-1: soluble triggering receptor expressed on myeloid cells-1; ICU: intensive care unit.

| **Variable** | | **Coefficient** | **Standard Error** | **Chi^2^** | **P value** | **Odds Ratio (95%CI)** |
| --- | --- | --- | --- | --- | --- | --- |
| SAPS II score | | 0.06 | 0.02 | 14.4 | 0.0001 | 1.07 (1.03-1.10) |
| SOFA score | | 0.03 | 0.07 | 0.17 | 0.67 | 1.03 (0.89-1.19) |
| Age | | 0.02 | 0.02 | 1.31 | 0.25 | 1.02 (0.98-1.06) |
| Body Temperature | | 0.03 | 0.21 | 0.02 | 0.87 | 1.03 (0.68-1.58) |
| Vasopressor use | | 0.61 | 0.75 | 0.66 | 0.42 | 1.84 (0.42-8.01) |
| Mechanical ventilation | | 1.69 | 0.95 | 3.14 | 0.08 | 5.44 (0.84-35.35) |
| PCT | Q2 | -0.19 | 0.78 | 0.06 | 0.80 | 0.82 (0.18-3.81) |
|  | Q3 | 1.07 | 0.69 | 2.39 | 0.13 | 2.92 (0.75-11.38) |
|  | Q4 | 0.14 | 0.75 | 0.03 | 0.85 | 1.15 (0.26-5.04) |
| sTREM-1 | Q2 | 0.27 | 0.74 | 0.14 | 0.71 | 1.31 (0.31-5.58) |
|  | Q3 | -0.17 | 0.76 | 0.05 | 0.82 | 0.84 (0.19-3.75) |
|  | Q4 | 2.08 | 0.78 | 7.20 | 0.007 | 8.04 (1.75-36.89) |

**Table S3.** Independent predictors of all-cause ICU mortality.

ICU: intensive care unit; sTREM-1: soluble triggering receptor expressed on myeloid cells-1; SAPS: simplified acute physiologic score; SOFA: sequential organ failure assessment; CI: confidence interval; PCT: procalcitonin
